# Chinese herbal compounds containing scorpion in the treatment of epilepsy: A protocol for systematic review and meta-analysis

**DOI:** 10.1097/MD.0000000000025134

**Published:** 2021-03-12

**Authors:** Ping Rong, Qianfang Fu, Xilian Zhang, Xuan Liu, Jie Yang, Xin Wang, Shuai Wang, Hui Liu, Rong Ma, Lianghui Nie, Rong Ma

**Affiliations:** a Department of Pediatrics, First Teaching Hospital of Tianjin University of Traditional Chinese Medicine, Xiqing; b National Clinical Research Center for Chinese Medicine Acupuncture and Moxibustion, Xiqing, Tianjin, China.

**Keywords:** epilepsy, meta-analysis, scorpion, systematic review, traditional Chinese medicine

## Abstract

**Background:**

Epilepsy is 1 of the common neurodevelopmental diseases. It can affect about 0.5% to 1.0% of the population regardless of their race and social class. Despite the development of a wide range of treatments, there remaining about one-third of patients still experience seizures. Chinese herbal compounds containing scorpion (CHCCS) have shown an outstanding curative effect on nerve protection and epilepsy. But there's no study to assess its clinical efficacy and safety.

**Methods:**

Each data of CHCCS in treating epilepsy from related English and Chinese databases will be searched. The primary outcome is the efficacy of the CHCCS on epilepsy. And the secondary outcomes include recurrence rate and side effects. The risk of bias will be assessed, and RevMan5.3 and Stata14.0 will be performed for meta-analysis. Finally, we will assess the level of the resulting evidence.

**Results:**

The results of the study will be combined with current evidence and published in a peer-reviewed journal.

**Conclusions:**

This study will specifically investigate the effectiveness and safety of CHCCS in treating epilepsy.

**INPLASY registration number:**

INPLASY202120056.

## 1 Introduction

Epilepsy is one of the common neurodevelopmental diseases. It is caused by an abnormal and sudden firing of neurons in the brain that causes temporary brain dysfunction.^[1]^ It is characterized by a persistent tendency to have seizures and may lead to cognitive impairments and even sudden death.^[2]^ It can affect about 0.5% to 1.0% of the population regardless of their race, age, economic status, social class, educational background, and geographical location.^[3,4]^ Despite the development of a wide range of treatments, such as anti-epileptic medication therapy and surgery, there remaining about one-third of patients still experience seizures.^[5]^ Except for the psychological consequences for the patients and their families, epilepsy and the potential for recurrence of epilepsy have also brought a large and increasing economic burden to society.^[6,7]^

The International League Against Epilepsy (ILAE) has clearly stated that the goal of anti-epileptic therapy is to control epileptic seizures completely, avoid side effects, and maintain a normal lifestyle.^[8]^ Although a lot of drugs are available for patients with epilepsy, their efficacy is limited. Overall, after reasonable and standardized anti-epileptic drug treatment, a part of epileptic patients can be effectively controlled, and there are nearly 30% of patients progressing to drug-resistant epilepsy.^[9,10]^ Besides, anti-epileptic drugs also bring some adverse effects. For example, a meta-analysis shows that carbamazepine has an adverse effect on bone health in people with epilepsy, and lacosamide can also lead to some adverse effects, such as headache, dizziness, diplopia, drowsiness, and cardiovascular abnormalities.^[11,12]^ Therefore, more effective and safe treatment has become the requirement of patients.

Traditional Chinese medicine has the advantage of preventing and treating epilepsy with better tolerance and lower cost over western medicine. Chinese herbal compounds containing scorpion (CHCCS), such as “spasmolytic powder” (a proportioned mixture of scorpion and centipede), “Qian-Zheng-San,” and “Xifeng Capsule” have shown an outstanding curative effect on nerve protection and epilepsy.^[13–15]^ However, it can also lead to some adverse effects, such as allergic reactions and liver injury.^[16]^ There's no study to assess its clinical efficacy and safety. Thus, this study will assess the effectiveness and safety of CHCCS in treating epilepsy.

## 2 Methods

### 2.1 Protocol register

This study has been registered at INPLASY (registration number: INPLASY202120056; https://inplasy.com/inplasy-2021-2-0056/). Our report is based on the guidelines of the preferred reporting items for systematic reviews and meta-analysis (PRISMA) Protocol statement.^[17,18]^

### 2.2 Eligibility criteria

#### 2.2.1 Types of studies

All clinical randomized controlled trials labeled with or without blind method will be included. If less than 5 papers are collected, we will broaden the research criteria to include non-randomized studies and semi-randomized control studies using the Cochrane Effective Practice and Organization of Care approach.^[19]^ The language of literature will be limited to English and Chinese. The animal study, conference papers, case reports, protocol, comments, supplementary issues, and reviews will be excluded.

#### 2.2.2 Types of patients

Patients with diagnosed epilepsy will be included, and there will be no restriction on the type of epilepsy. The patients enrolled regardless of their gender, age, race, and duration.

#### 2.2.3 Types of interventions and controls

The experiment group used the CHCCS regardless of dosage forms. Besides, CHCCS combined with other effective therapies will also be included. The control group applied for placebo, no cure, drug treatment, or other active therapies.

#### 2.2.4 Types of outcomes

The primary outcome is the efficacy of the CHCCS on treating epilepsy. And the secondary outcomes include recurrence rate and side effects.

#### 2.2.5 Electronic searches

We will search China National Knowledge Infrastructure (CNKI), Wan-fang database, VIP Database, Chinese Biomedical Literature Database (CBM), PubMed, Web of Science, EMBASE, Cochrane Library, International Clinical Trials Registry Platform. We will also search the Chinese Clinical Trial Registry Centers and Grey Literature. The retrieval time is limited from database establishment to February 2021. We will contact the author if there is a lack of essential data. Take PubMed as an example, the search strategy is summarized in Table 1.

**Table 1:** Search strategy used in PubMed database.

Number	Search terms
1	Randomized controlled trial
2	Clinical Trials, Randomized
3	Trials, Randomized Clinical
4	Controlled Clinical Trials, Randomized
5	or 1–4
6	Epilepsy
7	Epilepsies
8	Seizure Disorder
9	Seizure Disorders
10	Awakening Epilepsy
11	Epilepsy, Awakening
12	Epilepsy, Cryptogenic
13	Cryptogenic Epilepsies
14	Cryptogenic Epilepsy
15	Epilepsies, Cryptogenic
16	Aura
17	Auras
18	or 6–17
19	Medicine, Chinese Traditional
20	Traditional Chinese Medicine
21	Scorpion
22	or 19–21
23	5 and 18 and 22

#### 2.2.6 Searching other resources

We will also search the reference lists of meeting minutes and identified publications. The following literature established earlier than the database in China, such as “*China Journal of Traditional Chinese Medicine and Pharmacy*” and “*Journal of Traditional Chinese Medicine*” will also be searched.

### 2.3 Data management and analysis

#### 2.3.1 Selection of studies

**Figure 1:**
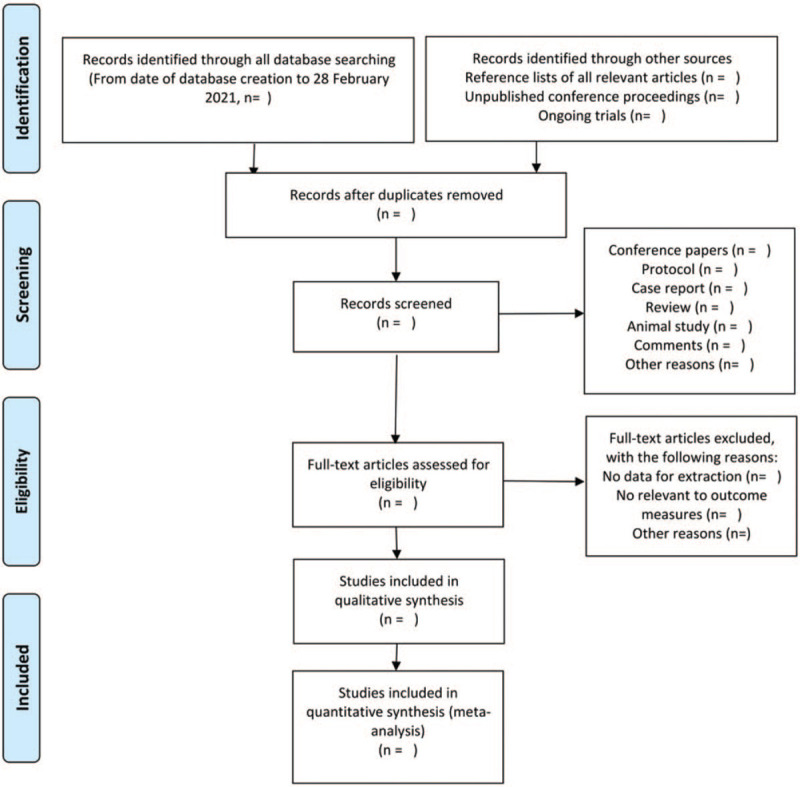
Flow diagram of studies identified.

We will import all retrieved literature into Endnote X9 and remove the duplicate studies. Firstly, two review authors (PR and QFF) will independently screen the titles and abstracts to exclude unrelated studies. Secondly, they will read the full texts carefully to further determine whether they fulfill all eligibility criteria as a second filtration. Eventually, the included studies will be crosschecked by two authors. Any divergence will be solved by a discussion with a third author (Dr. RM). The flow chart of selection details will be provided in Figure 1.

#### 2.3.2 Data extraction

The two authors (XLZ and XL) will input data independently using the data extraction form developed by the researchers. The selected trial information includes the basic information of the study (title, detailed information of the author, publication time, sources of funds), sample size, patients’ age, gender, diagnostic criteria, type of epilepsy, severity, the dose of the scorpion, course of treatment, grouping method, efficacy evaluation, and adverse effects.

#### 2.3.3 Risk of bias assessment

The Cochrane Collaboration's risk of bias tool will be adopted to assess the risk of bias. For each item, “low,” “unclear,” or “high” will be used as the key to the bias of risk. This work will be carried out by two authors (JY and XW). The third author (Dr. Ma) will make the final decision if disagreements appear.

#### 2.3.4 Statistical analysis

RevMan V.5.3.0 will be used to analyze the data. For dichotomous data, the risk ratio with a 95% confidence interval will be estimated. For continuous data, we will calculate the standardized mean difference or mean difference with 95% confidence intervals, depending on whether we use the same scale to measure an outcome in different studies. A *P*-value < .05 represents statistical significance. This work will be performed by two authors (SW and HL) independently to increase accuracy.

#### 2.3.5 Heterogeneity assessment

The Chi-squared and *I*^2^ tests will be calculated to assess the heterogeneity. When *P* > .1, *I*^2^ < 50%, considerable no heterogeneity in the included studies will be confirmed, and the fixed effect model will be applied. If not, a random effect model. We will apply subgroup analysis to investigate the potential clinical heterogeneity. We will perform a systematic review if we cannot find the source of heterogeneity.

#### 2.3.6 Subgroup analysis

According to the differences in the dosage form, dose of the scorpion, type of disease, course of treatment, and patients’ age, the subgroup analysis will be performed.

#### 2.3.7 Sensitivity analysis

The robustness of the results should be identified by performing the sensitivity analysis according to the following criteria: sample size, the quality of studies, missing data, and heterogeneity qualities.

#### 2.3.8 Publication bias

When studies included are more than 10, a funnel plot will be performed and we will assess whether it is symmetrical. If not, we will further perform an Egger test to reduce visual errors.^[20,21]^

#### 2.3.9 Quality assessment

We will perform the Grading of Recommendation Assessment, Development and Evaluation software to assess the quality of evidence for all outcomes.^[22,23]^

### 2.4 Ethics and dissemination

No ethical approval is inquired because nobody will be directly involved. If the protocol is modified, the information will be described in the final report.

## 3 Discussion

Epilepsy and seizures bring a great burden to the patients, their families, and society. The effectiveness of drug treatment is limited and there are some side effects. And there are nearly 30% of patients progressing to drug-resistant epilepsy. Therefore, effective and safe treatment has become the appeal of patients with epilepsy. CHCCS has shown good clinical efficacy, but there is no high-level evidence to assess its effectiveness and safety. So this study aims to assess it and provide convincing clinical evidence for clinicians.

## Author contributions

**Data curation**: Ping Rong, Qianfang Fu, Xilian Zhang, Xuan Liu.

**Formal analysis**: Jie Yang, Xin Wang, Shuai Wang, Hui Liu.

**Funding acquisition**: Ping Rong.

**Project administration**: Rong Ma.

**Supervision**: Rong Ma.

**Validation**: Ping Rong, Rong Ma.

**Writing – original draft**: Ping Rong, Qianfang Fu, Rong Ma, Lianghui Nie.

**Writing – review & editing**: Ping Rong, Qianfang Fu, Xilian Zhang.
